# Modified Xiaochaihu Decoction Combined with Mirtazapine in the Treatment of Persistent Depression: A Pilot Randomized Controlled Trial

**DOI:** 10.1155/2022/8682612

**Published:** 2022-06-27

**Authors:** Xin Li, Xiuyu Li, Man Gong, Chaoqun Li, Jing Li, Chao Zhou, Tingting He

**Affiliations:** ^1^The Second Ward of Liver Disease of Traditional Chinese Medicine, The Fifth Medical Center of Chinese PLA General Hospital, Beijing, China; ^2^Traditional Chinese Medicine Department, The Sixth Medical Center of Chinese PLA General Hospital, Beijing, China

## Abstract

**Background:**

Western drugs effectively manage persistent depressive disorder (PDD) but are associated with side effects.

**Objective:**

To observe the efficacy and safety of modified Xiaochaihu Decoction combined with mirtazapine in treating PDD.

**Methods:**

Patients with PDD were enrolled at the Naval General Hospital (06/2018–02/2019) and randomized to modified Xiaochaihu Decoction and modified Xiaochaihu Decoction with mirtazapine. The self-rating depression scale (SDS) and traditional Chinese medicine (TCM) scale were assessed at baseline and after 12 weeks. The overall clinical efficacy (primary outcome) and adverse reactions were observed.

**Results:**

Sixty-four participants completed the trial in the combined and control groups (30 and 28), respectively. In controls, the total effective rate was 78.6%, compared with 96.7% in the combined group (*P*=0.035). The scores of the SDS and TCM syndrome scale in the two groups were lower after treatment (*P* < 0.001) but without difference between groups (*P*=0.077). The combined group showed higher improvement rates regarding insomnia (96.4% vs. 44.0%, *P* < 0.001), bitter taste (90.5% vs. 52.6%, *P*=0.007), languid (72.0% vs. 31.8%, *P*=0.006), and belching/anorexia (100% vs. 52.6%, *P* < 0.001). The combined group showed a higher frequency of adverse events (73.3% vs. 3.6%) (*P* < 0.001).

**Conclusion:**

Modified Xiaochaihu Decoction combined with mirtazapine effectively treats PDD, and its curative effect is better than that of TCM alone.* Trial Registration.* This trial was registered with https://www.chictr.org.cn/index.aspx/ChiCTR2100048188.

## 1. Introduction

Persistent depressive disorder (PDD) is a disorder in which depressed mood occurs most of the time and lasts ≥2 years in adults or ≥1 year in adolescents and children [1, 2]. PDD is a Diagnostic and Statistical Manual of Mental Disorders, fifth edition (DSM-5) consolidation of dysthymia and chronic major depressive disorder (MDD). It has four possible presentations [3]: (1) PDD with the pure dysthymic syndrome (formerly dysthymia)—depressive symptoms for ≥2 years but criteria for MDD not met; (2) PDD with a persistent major depressive episode (formerly chronic major depressive disorder)—a persistent form (≥2 years of duration) of MDD that meets criteria for both PDD (dysthymia) and MDD; (3) PDD with an intermittent major depressive episode (formerly double depression)—the coexistence of both PDD and a concurrent episode of MDD (without chronic MDD); (4) PDD with intermittent major depressive episode without current MDD episode—PDD and history of MDD but MDD criteria are not currently met. The prevalence of PDD worldwide is estimated to be between 1% and 5% of children, adults, and the elderly [4–8]. Psychosocial factors may increase the risk of PDD, including acute loss (bereavement), substance abuse, childhood mistreatment, domestic abuse, traumatic events (such as car accidents), family history of depressive disorders, racial/ethnic minority status, and low socioeconomic status or financial difficulties [1, 2, 8].

The long course of PDD seriously affects their quality of life [9, 10]. Medication is still the primary treatment method [1, 11, 12]. Traditional Chinese medicine (TCM) can also be used to manage PDD [13–15]. Both TCM and western medicine have certain curative effects, but each has advantages and disadvantages [13, 16]. Simple western medication has stable and lasting effects. However, many patients can hardly persist or stop taking drugs due to the strong side effects. Although TCM's side effects are milder than western medicine, the effect onset is slow, and the effect cannot be stable and lasting [13–15].

Aung et al. [17] and Ye et al. [18] highlighted that TCM combined with western medicine could treat psychiatric diseases. Such an approach has milder side effects while ensuring a certain curative effect, thus increasing patients' compliance, improving the treatment outcomes, and improving the quality of life of the patients. Xiaochaihu Decoction is a TCM used to treat depression [19–23]. Mirtazapine is a tetracyclic antidepressant with an antagonist effect on central presynaptic *α*-2-adrenergic receptors, leading to increased serotonin and norepinephrine [24]. It is primarily used to treat MDD [24–26]. Side effects include drowsiness (54%), xerostomia (25%), and weight gain (12%) [24].

Therefore, this study aimed to observe the efficacy and safety of modified Xiaochaihu Decoction combined with mirtazapine in treating persistent depression of liver and kidney-yin deficiency type, suggesting novel methods to treat patients with PDD while optimizing their quality of life.

## 2. Materials and Methods

### 2.1. Study Design and Participants

Patients with PDD who visited the Outpatient Department of Traditional Chinese Medicine of the Naval General Hospital from June 2018 to February 2019 were consecutively approached for participation in this pilot randomized controlled trial. The Ethics Committee approved this study of the Sixth Medical Center of the People's Liberation Army General Hospital (No. 202104021). All participants signed the informed consent before entering the study.

The eligibility criteria were as follows: (1) the patients should meet the diagnostic criteria of PDD of the DSM-5 [3] and meet the diagnosis of “a deficiency of liver-yin and kidney-yin” of the Diagnosis and Treatment Standards of Traditional Chinese Medicine Diseases and Syndromes (2017 Edition) [27] and Clinical Epidemiological Questionnaire of Common Traditional Chinese Medicine Syndromes Standards of Depression [28] (depression or irritability, crying easily, sigh too much; burnout, fatigue, or soreness of waist and knees; dysphoria in chest-palms-soles, and hyperhidrosis, dry mouth and bitter taste, frequent belching or poor appetite, and insomnia. Emotional symptoms are necessary, and three of the other five symptoms are required. Organic diseases of other systems were excluded after various examinations); (2) Zung's self-rating depression scale (SDS) was ≥41 points [29]; (3) patients should have 20–69 years of age, regardless of gender; (4) they could correctly understand the content of the scales. The exclusion criteria were as follows: (1) patients who received other antidepressants within 2 weeks after the start of this study, (2) history of alcohol and drug dependence, (3) pregnant or lactating women, (4) uncontrolled chronic diseases such as hypertension and diabetes, (5) abnormal liver and kidney function, (6) fever, pneumonia, trauma, or other diseases at screening, (7) confirmed or suspected bipolar disorder, or (8) patients with suicidal tendencies or experiences. The participants who quit after entering the study, had poor compliance, or failed to carry out examination and treatment according to the protocol were excluded from the analyses.

### 2.2. Grouping

The participants were divided into the Chinese medicine control group (modified Xiaochaihu Decoction) and the Chinese and western medicine combined treatment group (modified Xiaochaihu Decoction combined with mirtazapine) by the random number table method.

### 2.3. Interventions

The traditional Chinese medicine control group was treated with the modified Xiaochaihu Decoction, one dose a day separated into two parts (morning and evening), made from 15 g of *Bupleurum*, 15 g of *Pinellia ternata*, 15 g of *Codonopsis pilosula*, 6 g of roasted licorice, 10 g of ginger, 15 g of *Scutellaria baicalensis* Georgi, 10 g of jujube, 30 g of lily, 15 g of Rehmannia root, and 30 g of blighted wheat.

The combined treatment group was treated with the same modified Xiaochaihu decoction as the control group and was treated with mirtazapine (Organon International, Oss, The Netherlands). The participants took 15 mg of mirtazapine once every night on the 1st-4th day, 1 h before going to bed, and 30 mg once every night starting on the 5th day and after that, 1 h before going to bed.

### 2.4. Observation Index

The main indicator of observation is the effectiveness after treatment. The participants were scored using the SDS and TCM syndrome scale before treatment and at the end of the 12th week of treatment, referring to the standards formulated in the Diagnosis and Treatment Standards of Traditional Chinese Medicine Diseases and Syndromes (2017 Edition) [27] and Guiding Principles for Clinical Research of New Drugs of Traditional Chinese Medicine [30]. The clinical curative effect was divided into four grades: clinical cure, remarkable effect, improvement, and ineffectiveness. The SDS scale (the total score range is 20–80 points, and the higher the score, the more serious the degree of depression) and the TCM syndrome scale (Supplementary Table S1) were used for scoring. SDS score reduction rate [31] = (pretreatment score - posttreatment score)/(pretreatment score - the lowest score of the scale) × 100%.

Clinical cure was defined as the disappearance of the chief complaint and accompanying symptoms, the mood was normal, and social function recovered; SDS score reduction rate was ≥80%. The remarkable effect was defined at the chief complaint, and accompanying symptoms mostly disappeared, and the mood was stable; the SDS score was reduced by 50%–80%. Improvement was defined as the partial disappearance of the chief complaint; SDS was reduced by 30%–50%. Ineffectiveness was defined as no significant improvement in symptoms and emotions; SDS was reduced by <30%.

The secondary outcome was the improvement of the patient's accompanying symptoms, judging from the patient's chief complaint symptoms during the follow-up visit. Pain is felt in the whole body, joints, and other parts (excluding other organic or functional diseases). Insomnia includes falling asleep or sleep maintenance disorder, affecting daytime life, work, and study functions. Bitter taste was defined as always feeling a bitter taste in the mouth. Irregular stool included abnormal stool texture and abnormal stool frequency. Burnout and fatigue included listlessness, decreased activity or labor endurance, and even inability to carry out routine daily work or study. Belching and anorexia included symptoms of dyspepsia, loss of appetite, or frequent sounds from the throat caused by gas in the stomach, which are long and slow. Hyperhidrosis was defined as an abnormal increase in local or systemic sweating.

### 2.5. Safety Assessment

The Treatment Emergent Symptom Scale (TESS) was used to evaluate the safety of the participants in both groups [32]. The scores of TESS were evaluated after treatment.

### 2.6. Follow-Up

Telephone or outpatient follow-up was conducted once every two weeks.

### 2.7. Statistical Analysis

According to the mechanism of missing data, that is, missing completely at random (MCAR), missing at random (MAR), and missing not at random (MNAR), the methods of ignoring missing value, filling method, intention analysis, and sensitivity analysis were adopted, based on the Specification for Statistical Processing and Reporting of Falling-off, Withdrawing, and Lost-follow Cases in Clinical Trials [33].

SPSS 20.0 (IBM, Armonk, NY, USA) was used for statistical analysis. The continuous data were tested for normal distribution using the Shapiro-Wilk test. The continuous data are presented as means ± standard deviations and were analyzed using Student's *t*-test (intergroup comparisons) or the paired *t*-test (intragroup comparisons). The categorical data are expressed as *n* (%) and were analyzed using the chi-square test. Two-sided *P* values <0.05 were considered statistically significant.

## 3. Results

### 3.1. Baseline Characteristics of the Two Groups of Patients

**Figure 1:**
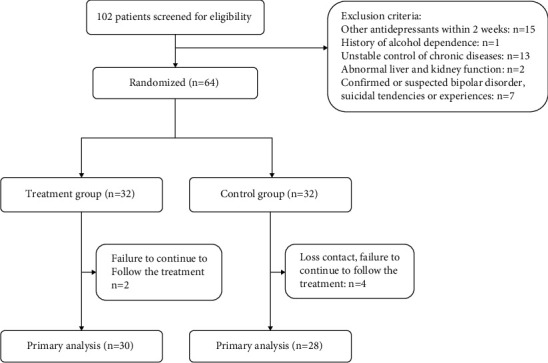
Participant flowchart.

Sixty-four PDD participants were enrolled (Figure 1), with a course of PDD of 24–120 months (2–10 years). Their characteristics are shown in Table 1. There were 32 participants in the treatment group. The mean age was 47.3 ± 12.6 (range: 21–69) years, including 13 males and 19 females. The mean disease course was 3.5 ± 1.5 (range: 2–7) years. There were 32 participants in the control group. The mean age was 45.4 ± 9.3 (range: 22–66) years old, including 11 males and 21 females. The longest disease course was 7 years, and the shortest was 2 years, with an average of 3.6 ± 1.6 years. There were no significant differences between the two groups (*P* > 0.05) (Table 1).

### 3.2. The Effective Rate between the Two Groups after Treatment

In the combined group, dropout was seen in two of 32 participants (the remaining 30 participants). In the control group, dropout was seen in four participants (the remaining 28 participants).

In the control group, two participants were cured (17.9%), nine were markedly effective (32.1%), 11 were improved (39.3%), and the total effective rate was 78.6%. In the combined group, 10 participants were cured (33.3%), 11 were markedly effective (36.7%), and eight were improved (26.67%), with a total effective rate of 96.7% (Table 2). The effective rate of the combined group and curative effect of the combined group was significantly better than in the control group, and they were also significantly better than in the control group (*P* < 0.05).

### 3.3. SDS and TCM Syndrome Scale Scores between the Two Groups

After 12 weeks of treatment, the SDS and TCM syndrome scale scores in the two groups were significantly lower after treatment than before (all *P* < 0.001). At the end of the 12th week, the scores of TCM syndromes in the combined group were 13.7 ± 2.02 points, while those in the control group were 14.6 ± 1.5 points (*P* < 0.05) (Table 3). There were no significant differences in SDS scores between the combined and control groups (30.8 ± 4.8 vs. 32.1 ± 3.8, *P* > 0.05).

### 3.4. Improvement of Concomitant Symptoms

In addition to emotional symptoms, patients in the two groups showed pain, tiredness, fatigue, insomnia, hyperhidrosis, irregular stool, dry mouth, bitter taste, anorexia, belching, and irregular stool (Table 4). After 12 weeks of treatment, both groups had improvements in the symptoms, and the combined group showed significantly higher improvement rates than the control group regarding insomnia (96.4% vs. 44.0%, *P* < 0.001), bitter taste (90.5% vs. 52.6%, *P* < 0.001), languid (72.0% vs. 31.8%, *P* < 0.001), and belching and anorexia (100% vs. 52.6%, *P* < 0.001) (Table 4).

### 3.5. Safety

After treatment, the scores of TESS were evaluated in both groups. The control group was 5.9 ± 1.1 points, and the combined group was 9.4 ± 2.6 points (*P* < 0.05), mainly due to the weight gain caused by mirtazapine tablets in the combined group (19 patients (63.3%) in the treatment group at 12 weeks). After taking mirtazapine, they had a slight increase in weight (2–5 kg), two patients had lethargy symptoms (6.7%), and one patient had tremors (3.3%). The control group showed only one participant with weight gain. The difference in all adverse events between the two groups was significant (73.3% vs. 3.6%) (*P* < 0.001) (Table 5).

## 4. Discussion

Western drugs effectively manage PDD but are associated with side effects and decreased quality of life [13–15]. TCM can be combined with western drugs for depressive disorders [17, 18]. This study observed the efficacy and safety of modified Xiaochaihu Decoction combined with mirtazapine in treating PDD. The results suggest that the modified Xiaochaihu Decoction combined with mirtazapine effectively treats PDD, and its curative effect is better than that of TCM alone. However, the combined group showed a higher frequency of adverse events.

In this trial, the dropouts represented 6.3% in the combined group and 12.5% in the control group, suggesting the general tolerability of the treatment. After 12 weeks of treatment, both groups achieved certain therapeutic effects. Still, the total clinical effective rate, cure rate, and obvious effective rate of the treatment group were significantly better than those of the control group. Studies have shown that the beneficial effects of the combined approach have been observed, but western medicine and TCM regimens are different. [17, 18]. PDD treatment with integrated TCM and western medicine in this pilot trial had advantages over the simple TCM group. In improving specific accompanying symptoms, the combined group showed more significant therapeutic effects on burnout, fatigue, insomnia, and anorexia than the TCM treatment group. Nevertheless, the TCM group showed some effects, especially in the improving effect of Xiao Chai Hu Tang on depression [15, 19, 21, 22].

Significant differences in adverse events between the two groups were mainly related to the effect of mirtazapine on appetite and body weight [24–26]. Still, at the same time, the combined group had significant advantages in improving digestive tract symptoms such as anorexia and belching. Still, gaining too much weight can negatively impact the quality of life and might have to be controlled using diet and exercise. Future studies should examine the impact of the baseline weight on weight gain and management of symptoms, which could not be done here because of the small sample size. The follow-up found that the weight can be kept stable after proper control of calorie intake and exercise, so mirtazapine is more suitable for people with a thin physique.

In the two groups of patients, insomnia, belching and anorexia, pain, burnout and fatigue, hyperhidrosis, and irregular stool were selected. It was found that insomnia (91.4%) accounted for the largest proportion of the above symptoms, followed by burnout and fatigue (81.0%), belching and anorexia (70.7%), bitter taste (dry mouth) (69.0%), and pain (63.8%). They suggest that nonpsychiatrists should consider the possibility of anxiety/depression disorder when treating “refractory” diseases complained of by the above symptoms on the premise of excluding organic conditions. At the same time, the study found unique lingual signs of depressive disorder. Among 58 patients, there were 53 patients with insomnia. Among them, the middle sulcus tongue was visible (Supplementary Figure S1). It is suggested that PDD, insomnia, and median sulcus of the tongue might be related. This phenomenon can be explained from the perspective of TCM. According to TCM theory, the tongue can reflect the rise and fall of qi and blood in the human body. The median sulcus of the tongue represents the performance of tongue dryness and demonstrates the degree of yin and blood loss in the body. TCM says that “Sleeping can nourish essence,” which indicates that sleep at night is the best time for the human body to nourish essence and explain the important relationship between sleep and yin and fluid. Therefore, insomnia patients have insufficient yin. When yin and blood are not nourished, it is reflected in the tongue.

This study had limitations. First, this trial lacked a western medicine control group. Because of practical factors, most patients who visit the outpatient departments of TCM in general hospitals seek TCM therapy, so it is difficult to establish a western medicine treatment group. Second, for PDD, the disease course varies from 2 to 20 years, and some patients even reach more than 30 years. Whether the improvement rate of patients is related to the course of the disease was not examined in this study. Finally, due to the inconvenience of outpatient management and poor communication with patients, some patients dropped out, and the test indicators were not collected completely.

## 5. Conclusions

In conclusion, the effect of modified Xiaochaihu Decoction combined with mirtazapine is better than TCM alone in treating PDD, which provides guidance and reference for future clinical work.

## Figures and Tables

**Table 1:** Baseline characteristics of the participants.

Characteristics	Combined group (*n* = 32)	Control group (*n* = 32)	*P*
Age, years (mean ± SD)	47.3 ± 12.6	45.4 ± 9.3	0.499
Male/female	13/19	11/21	0.595
Course of disease, years (mean ± SD)	3.5 ± 1.5	3.6 ± 1.6	0.793
Insomnia, dreaminess, *n* (%)	28 (87.5)	25 (78.1)	0.223
Belching and anorexia, *n* (%)	22 (68.8)	19 (59.4)	0.434
Languid, *n* (%)	25 (78.1)	22 (68.8)	0.396
Dry mouth, bitter mouth, *n* (%)	21 (65.6)	19 (59.4)	0.606

**Table 2:** The effective rate between the two groups after treatment.

Characteristics, *n* (%)	Combined group (*n* = 30)	Control group (*n* = 28)	*P*
Ineffective	1 (3.3)	6 (21.4)	0.035
Effective	29 (96.7)	22 (78.6)	
Cure	10 (33.3)	2 (17.9)	0.014
Marked effect	11 (36.7)	9 (32.1)	
Improved	8 (26.7)	11 (39.3)	

**Table 3:** SDS and TCM syndrome scores between the two groups before and after treatment.

Characteristics (mean ± SD)		Combined group (*n* = 30)	Control group (*n* = 28)	*P*
Before treatment	TCM syndrome score	23.1 ± 2.6	20.5 ± 2.1	<0.001
	SDS scale score	51.1 ± 1.4	43.1 ± 0.3	<0.001

After treatment	TCM syndrome score	13.7 ± 2.0	14.6 ± 1.5	0.077
	SDS scale score	30.8 ± 4.8	32.1 ± 3.8	0.255
	Score difference of TCM syndrome scale	9.4 ± 3.1	5.9 ± 1.4	<0.001
	Score difference of SDS scale	20.3 ± 8.5	28.5 ± 2.0	<0.001

SDS: Zung's self-rating depression scale; TCM: traditional Chinese medicine.

**Table 4:** Improvement of accompanying symptoms in the two groups.

Symptoms	Combined group		Control group		*P*
	Before/after	Improvement rate (%)	Before/after	Improvement rate (%)	
Pain	20/8	60.0	17/12	29.4	0.063
Insomnia/dreaminess	28/1	96.4^a^	25/14	44.0	<0.001
Bitter taste (dry mouth)	21/2	90.5^b^	19/9	52.6	0.007
Irregular stool (loose stool/constipation)	16/4	75.0	14/4	71.4	0.825
Languid	25/7	72.0^c^	22/15	31.8	0.006
Belching and anorexia	22/0	100^d^	19/9	52.6	<0.001
Hyperhidrosis	14/3	78.6	12/2	71.4	0.759

^a^ *P*=0.001, ^b^*P*=0.007, ^c^*P*=0.006, ^d^*P*=0.001.

**Table 5:** Adverse events after treatment.

Characteristics, *n* (%)	Combined group (*n* = 30)	Control group (*n* = 28)	*P*
Adverse events	22 (73.3)	1 (3.6)	<0.001
Drowsiness	2 (9.1)	0	
Tremor	1 (4.5)	0	
Weight gain	19 (86.4)	1 (100.0)	

## Data Availability

The data set supporting the results of this article are included within the article. The datasets used and/or analyzed during the current study are available from the corresponding author on reasonable request.

## Supplementary Materials

Supplementary Materials Supplementary Table S1: classification and quantitative table of TCM syndromes of deficiency of liver-yin and kidney-yin. Supplementary Figure S1: the manifestation of “median sulcus of the tongue” in patients with persistent depressive disorder.
