# Supplementary material for: Modified Xiaochaihu Decoction Combined with Mirtazapine in the Treatment of Persistent Depression: A Pilot Randomized Controlled Trial
Source: Contrast Media Mol Imaging. 2022 Jun 27;2022:8682612. doi: 10.1155/2022/8682612 (PMC9252686; doi:10.1155/2022/8682612)
Supplement: Supplementary Materials — Supplementary Table S1: classification and quantitative table of TCM syndromes of deficiency of liver-yin and kidney-yin. Supplementary Figure S1: the manifestation of “median sulcus of the tongue” in patients with persistent depressive disorder. [file 8682612.f1.doc]

**Supplementary Table S1. Classification and quantitative table of TCM syndromes of deficiency of Liver-Yin and Kidney-Yin**

| Symptoms | Scoring standard | |
| --- | --- | --- |
| Depressed and unhappy | None | 0 point |
|  | Mild | 2 points |
|  | Moderate | 4 points |
|  | Severe | 6 points |
| Burnout, fatigue, or soreness of waist and knees | None | 0 point |
|  | Mild | 2 points |
|  | Moderate | 4 points |
|  | Severe | 6 points |
| Dry mouth, bitter taste | None | 0 point |
|  | Mild | 2 points |
|  | Moderate | 4 points |
|  | Severe | 6 points |
| Dysphoria in chest-palms-soles and hyperhidrosis | None | 0 point |
|  | Mild | 2 points |
|  | Moderate | 4 points |
|  | Severe | 6 points |
| Frequent belching or lack of diet | None | 0 point |
|  | Mild | 2 points |
|  | Moderate | 4 points |
|  | Severe | 6 points |
| Insomnia/dreaminess, or feeling unsatisfied with sleep | None | 0 point |
|  | Mild | 2 points |
|  | Moderate | 4 points |
|  | Severe | 6 points |

The tongue and pulse were recorded in detail without scoring.

None means that there has never been or very little, and it does not affect normal life and work. Mild refers to a small part of the time with little impact on life and work. Moderate refers to a considerable amount of time and affects work and life. Severe refers to the vast majority of the time or all the time, seriously affecting work and life, or even unable to work and live normally.


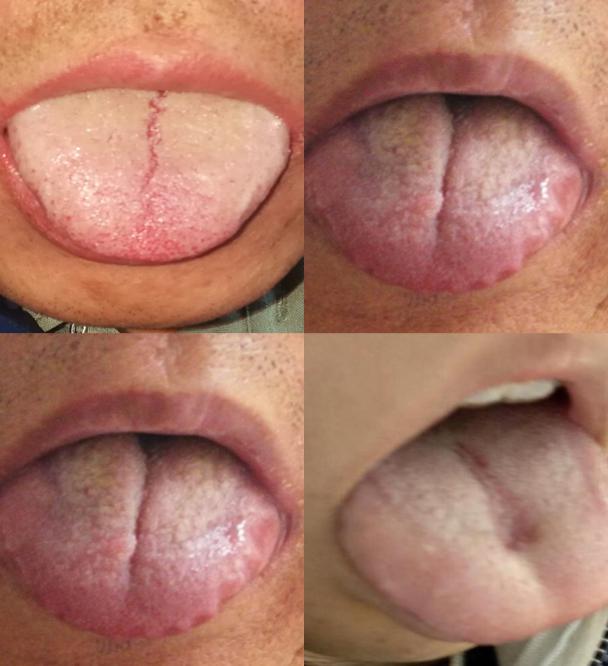


**Supplementary Figure S1.** The manifestation of “median sulcus of the tongue” in patients with persistent depressive disorder.
